# Trefoil Factor Family (TFF) Peptides and Their Diverse Molecular Functions in Mucus Barrier Protection and More: Changing the Paradigm

**DOI:** 10.3390/ijms21124535

**Published:** 2020-06-25

**Authors:** Werner Hoffmann

**Affiliations:** Institute of Molecular Biology and Medicinal Chemistry, Otto-von-Guericke University Magdeburg, Leipziger Str. 44, 39120 Magdeburg, Germany; werner.hoffmann@med.ovgu.de

**Keywords:** gastric cancer, reactive oxygen species, reactive nitrogen species, inflammation, trefoil factor, lectin, gastrokine, FCGBP, mucin, innate immunity

## Abstract

Trefoil factor family peptides (TFF1, TFF2, TFF3) are typically co-secreted together with mucins. *Tff1* represents a gastric tumor suppressor gene in mice. TFFs are also synthesized in minute amounts in the immune and central nervous systems. In mucous epithelia, they support rapid repair by enhancing cell migration (“restitution”) via their weak chemotactic and anti-apoptotic effects. For a long time, as a paradigm, this was considered as their major biological function. Within recent years, the formation of disulfide-linked heterodimers was documented for TFF1 and TFF3, e.g., with gastrokine-2 and IgG Fc binding protein (FCGBP). Furthermore, lectin activities were recognized as enabling binding to a lipopolysaccharide of *Helicobacter pylori* (TFF1, TFF3) or to a carbohydrate moiety of the mucin MUC6 (TFF2). Only recently, gastric TFF1 was demonstrated to occur predominantly in monomeric forms with an unusual free thiol group. Thus, a new picture emerged, pointing to diverse molecular functions for TFFs. Monomeric TFF1 might protect the gastric mucosa as a scavenger for extracellular reactive oxygen/nitrogen species. Whereas, the TFF2/MUC6 complex stabilizes the inner layer of the gastric mucus. In contrast, the TFF3–FCGBP heterodimer (and also TFF1–FCGBP) are likely part of the innate immune defense of mucous epithelia, preventing the infiltration of microorganisms.

## 1. Introduction

**Figure 1:**
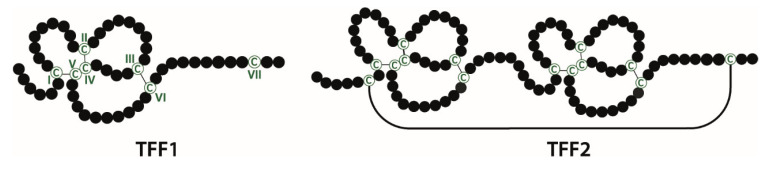
Disulfide-linked three-leafed structures of the prototypic trefoil factor family peptides TFF1 and TFF2. Cysteine residues (C) are shown in green (numbering in Roman numerals).

Mammalian trefoil factor family (TFF) peptides (TFF1, TFF2, and TFF3) are characterized by a common structural motif, the TFF domain (formerly: P-domain, trefoil domain), which contains six conserved cysteine residues with three intramolecular disulfide bonds (Cys^I-V^, Cys^II-IV^, and Cys^III-VI^; Figure 1). TFF1 (human: 60 amino acids) and TFF3 (human: 59 amino acids) consist of single TFF domains and additionally contain free 7^th^ cysteine (Cys^VII^) residues located outside the TFF domain. TFF2 (human: 106 amino acids) contains two TFF domains and the N- and the C-terminals are disulfide-linked via two additional cysteine residues outside the TFF domains (reviews: [1,2,3,4,5,6]).

In the skin of frogs, such as *Xenopus laevis*, even more TFF peptides exist [3]. Furthermore, shuffled TFF modules are present in a number of mosaic proteins, e.g., the human zona pellucida proteins ZP1 and ZPB, the sugar-degrading enzymes sucrase–isomaltase, α-glucosidase, and maltase–glucoamylase [3], the frog skin proteins APEG [7] and “βγ-crystallin and trefoil factor” (βγ-CAT) [8], and the frog integumentary mucins FIM-A.1 and FIM-C.1 [9,10,11]. Generally, TFF domains are encoded by single exons belonging to the class 1-1 and thus represent a unique family of cysteine-rich shuffled modules [3].

### 1.1. Exocrine and Endocrine Secretion of TFF Peptides

Mucous epithelia are the predominant expression sites of TFF peptides. Here, the exocrine secretion of TFFs occurs together with different mucins [3,6]. TFF1 and TFF2 are predominantly synthesized in the stomach, i.e., in surface mucous cells (SMCs) and mucous neck/antral gland cells, respectively [2,6]. Additionally, TFF2 is expressed in duodenal Brunner glands, and in some species, (such as the pig and mouse, but not human) also in the exocrine pancreas [1,12]. In contrast, TFF3 shows a much wider distribution and is found mainly in intestinal goblet cells, but also in the respiratory and urogenitary tracts, the salivary glands, esophageal submucosal glands, conjunctiva, and the inner ear (Table 1) [3,13,14]. Thus, TFFs appear in many body fluids, such as saliva, gastric juice, urine, tears, and breast milk [5].

Furthermore, minute amounts of TFF peptides are also secreted in an endocrine manner, e.g., by the central nervous system (CNS), as well as by the immune system (Table 1). In particular, TFF3 is synthesized in neurons (such as oxytocinergic neurons of the hypothalamus), activated microglial cells, and astrocytes of the brain [15,16,17,18]. All three TFF peptides show developmental changes, particularly in the cerebellum, the most pronounced being TFF3 [19]. In contrast, TFF2 (and to some extent also TFF3) is expressed in peritoneal macrophages and lymphoid tissues, such as the spleen (memory T-cells), thymus, lymph nodes, and bone marrow [20,21,22,23,24]. TFF3 (and little TFF1) is also expressed in the endocrine pancreas [25] and TFF3 is also expressed in the thyroid [26]. This would explain why all three TFF peptides are detectable in human serum [27].

### 1.2. Ectopic Expression of TFF Peptides during Inflammatory Conditions and in Tumors

TFF peptides, together with epidermal growth factor (EGF), are aberrantly expressed in diverse chronic ulcerative conditions, often in glandular structures termed the “ulcer-associated cell lineage” (UACL) [28]. Thus, TFF peptides are part of a chronic inflammatory response during a variety of diseases, such as ulcerative colitis, diverticulitis, cholecystitis, inflammatory bowel disease, gastro-esophageal reflux disease, pancreatitis, and chronic obstructive pulmonary disease [2,3,5,29,30,31,32].

Of special note, TFF1 in particular is ectopically expressed in various chronic inflammatory conditions, even in murine models of encephalitis and asthma, and in the murine spleen after *Toxoplasma gondii* infection [31,33,34,35,36].

Further pathological expression of TFF peptides occurs in metaplasias [37], as well as in different kinds of tumors [2,3,5,38,39]. Of note, somatic mutations in the TFF1 gene seem to be associated with gastric cancer and there is a strikingly reduced TFF1 expression in the majority of gastric carcinomas [40,41].

### 1.3. Phenotypes of Tff-Deficient (Tff^KO^) Animals

For a long time, mice deficient in *Tff1*, *Tff2* and *Tff3* have been available. The most prominent phenotype is observed in *Tff1*^KO^ mice. 

*Tff1*^KO^ mice mainly show a gastric phenotype, i.e., they all develop antropyloric adenomas with ~30% progressing to carcinomas [42,43]. Thus, these mice represent a recognized model for spontaneous antral tumorigenesis [44]. Of note, *Tff1*^KO^ mice also show strongly reduced Tff2 expression, particularly in the gastric corpus [42,45]. Antral carcinogenesis is an age-dependent multi-step process and is accompanied by NF-κB-mediated chronic inflammation [46,47]. Tumor growth can be suppressed with the selective Cox-2 inhibitor celecoxib [43,47,48]. There are indications that long-lived precursor cells in antral units located at positions +4 (*Cckbr*^+^) and +5 (*Mist*^+^) are involved in tumorigenesis in *Tff1*^KO^ mice [45]. Of note, in *Tff1*^KO^ mice, continuous self-renewal is also dysregulated in fundic units, but this does not lead to carcinogenesis [49]. Furthermore, *Tff1*^KO^ mice show generally increased chemically-induced tumorigenesis [50]. Taken together, *Tff1* represents a gastric tumor suppressor gene in mice [43] and TFF1 mutations and dysregulated TFF1 expression seem to be critical to the pathogenesis of most gastric carcinomas in humans [40,41]. Of note, mutations in the interleukin 6 (IL6) signal transducer gp130, which blocked SHP2–Ras–ERK signaling (*gp130*^757F^ mice), showed reduced Tff1 levels and a phenotype (antropyloric adenomas, but no carcinomas) highly similar to *Tff1*^KO^ mice [51,52].

In contrast, *Tff2*^KO^ mice do not show a striking phenotype. Other than moderate morphological changes [53], they have a highly increased Tff3 expression in the antrum [54]. They also have increased susceptibility to *Helicobacter*-induced gastritis and to *Yersinia enterocolitica* infection, as well as a delayed recovery after dextran sodium sulfate (DSS)-induced colitis [22,55,56,57]. Furthermore, *Tff2*^KO^ mice show the dysregulated expression of immune response-related genes and their macrophages are hyperresponsive to interleulin-1β [22,54].

Nine-day-old (P9) *Tff2*^KO^ rats are highly sensitive to oral infection with *Escherichia coli* K1, leading to bacteremia probably due to the loss of Tff2 in the developing small intestine [58]. Of note, *Tff2* expression is developmentally regulated in the neonatal rat intestine, reaching a peak at P9 and dropping sharply thereafter [59].

*Tff3*-deficient mice have strongly increased sensitivity in the DSS colitis model and these animals are particularly sensitive to radiation-induced mucosal injury and chemotherapy [60,61]. Of note, mutations in the interleukin 6 (IL6) signal transducer gp130, which abrogated STAT1/3 signaling (*gp130*^ΔSTAT^ mice), showed reduced Tff3 levels and a phenotype highly similar to *Tff3*^KO^ mice [51,52]. Furthermore, *Tff3*^KO^ mice show minor morphological differences in the foliation of the cerebellum, as well as motoric deficits [62].

### 1.4. TFF Peptides Enhance Cell Migration: Implications for Mucosal Protection and Immune Responses

There is a large body of literature describing the weak motogenic effects of all three TFF peptides for a variety of different epithelial and immune cells in vitro, starting in the year 1994 [3,5,63]. Thus, relatively rapidly, this became a paradigm explaining the biological function of TFF peptides with no clear differences between TFF1, TFF2, and TFF3. For example, TFFs enhance the cell migration of intestinal epithelial cells [63,64], corneal epithelial cells [65,66], bronchial epithelial cells [67], gastric epithelial cells [68], oral keratinocytes [69], pancreatic cells [70], and monocytes [20]. All three TFF peptides show generally weak chemotactic activity [71,72]. There is also a synergistic effect with epidermal growth factor (EGF) [72,73]. In line with their motogenic effects, TFF peptides were also described as pro-angiogenic factors [74].

For all three TFF peptides, an anti-apoptotic effect has also been observed [75,76,77,78]. However, for TFF3, a pro-apoptotic effect was also reported [79]. A combined motogenic and anti-apoptotic activity of TFF peptides would be in line with the observation that, during invasion of the extracellular matrix, cell migration and survival mechanisms are coordinately regulated [80]. This ensures that both effects synergistically support the important process of cell migration/invasion, e.g., during wound healing, immune responses, and angiogenesis.

A special form of rapid wound healing occurs in mucous epithelia after superficial injury, i.e., by the cell migration of neighboring cells. This process is called “restitution” and starts within minutes after damage. Thus, TFFs would be well designed to act as luminal protection peptides, enhancing restitution only after mucosal injury [81,82].

A protective function of TFF peptides for mucous epithelia is in line with numerous in vivo studies using different animal models of disease (after chemical damage or restraint; for compilations, see [3,5]). Additionally, the active delivery of TFF peptides by genetically modified *Lactococcus lactis* was effective in preventing and healing DSS-induced colitis [83].

TFF3 is also linked to innate immunity, as its synthesis in intestinal goblet cells is selectively induced after the activation of Toll-like receptor (TLR) 2 by commensal bacteria [84]. This probably occurs by an indirect mechanism. Of note, recombinant TFF3 can rescue *Tlr2*^KO^ mice from increased morbidity and mortality during acute colonic injury, probably due to its anti-apoptotic effect [84].

Generally, the motogenic and anti-apoptotic effects of TFF peptides are rather weak and were observed in a concentration range of about 10^−6^ to 10^−7^ M or even higher [13,71]. Such relatively high concentrations are atypical of a classical high-affinity peptide ligand, such as epidermal growth factor (EGF), which activates its specific receptors even at concentrations as low as 10^−10^ M [72]. Thus, it is not surprising that the repeated attempts to identify high-affinity receptors for TFF peptides have failed [85].

However, several transmembrane proteins could be characterized to bind TFF2 with low affinity, such as a porcine β-integrin and a porcine 224k protein (CRP-ductin), with similarity to *Deleted in Malignant Brain Tumors* 1 (DMBT1) [86]. In addition, TFF3 has been reported to bind to the secreted variant DMBT1^gp340^ in a Ca^2+^-dependent manner [87], the latter being an agglutinin playing a role in mucosal innate immunity [88]. Later on, TFF2 and TFF3 were described as low-affinity ligands for the chemokine receptors CXCR4 and CXCR7, i.e., TFF2 and TFF3 were active at a concentration of about 5 × 10^−7^ M [89,90]. Thus, the ligation of TFF peptides to CXCR4 and CXCR7 would explain their chemotactic effects, as CXCR4 and CXCR7 are the high-affinity receptors for the chemokine CXCL12/stromal cell-derived factor (SDF-1), which is a highly potent chemotactic peptide and regulates apoptosis at a concentration below 10^−9^ M [91]. Thus, to some extent, the TFF2-CXR4 axis in particular seems to play a direct role in gastric repair [92], as well as in suppressing colorectal carcinogesis via the neural innervation of splenic memory T-cells [24]. Furthermore, cell migration was also promoted by TFF2 (2 × 10^−7^ M) via the activation of the proteinase-activated receptor PAR4 [93], and TFF3 (10^−6^ M) was claimed to activate PAR2 [94].

Taken together, TFF peptides cannot be considered as high-affinity ligands for specific transmembrane receptors. As their concentrations in mucous gels are rather high, it seems highly unlikely that their protective function is based solely on their action as low-affinity ligands, e.g., for CXCR4 and CXCR7. It is more realistic to expect additional molecular functions for TFF peptides.

## 2. Molecular Forms of TFF Peptides and Their Interaction Partners: Functional Implications

TFF1-3 are typical secretory peptides. TFF1 and TFF3 are special, as they contain an odd number of cysteine residues, with Cys^VII^ located outside the conserved TFF domain. Generally, the existence of unpaired cysteine residues is highly unlikely for secretory proteins, as disulfide formation is enforced in the endoplasmic reticulum (ER) [95]. Thus, TFF1 and TFF3 were expected to occur naturally as homodimers and most of the in vitro wound healing experiments and the in vivo studies using animal models were indeed performed with homodimers. There are reports that the dimeric forms are biologically more active than the monomeric forms [71,96,97,98]. Even the binding of TFF1 to *Helicobacter pylori* and TFF3 to DMBT1^gp340^ was reported to depend on dimerization [87,99].

Only later did biochemical studies reveal that TFF1 and TFF3 occur in vivo in different molecular forms and are also capable of forming disulfide-linked heterodimers with at least gastrokine 2 (GKN2) and IgG Fc binding protein (FCGBP). Thus far, the following hetero(di)meric forms have been identified: TFF1-GKN2, TFF1-FCGBP, and TFF3-FCGBP [45,100,101,102,103]. This indicates that the biological function of TFF peptides is obviously more complex.

### 2.1. TFF Domains Have Different Lectin Activities

All three TFF peptides were shown to bind to carbohydrate moieties, but to quite different extents and probably also with different specificities. Dimeric TFF1 interacts with a core oligosaccharide portion of the *H. pylori* lipopolysaccharide in a pH-dependent manner and TFF3 binds to a lesser extent [99,104,105]. Binding studies with truncated lipopolysaccharides from *H. pylori* mutants point to N-acetylglucosamine (GlcNAc) being part of the carbohydrate structure recognized by TFF1 [105]. Dimeric TFF1 also binds weakly to the gastric mucin MUC6, probably to a carbohydrate moiety conserved from frog to human, e.g., the terminal αGlcNAc residue (see also Section 2.3) [103,105]. TFF2 is strongly, but non-covalently, bound to gastric mucus [12,106]; the specific Ca^2+^- and pH-dependent lectin activity of TFF2 was narrowed down to the unusual O-linked terminal carbohydrate moiety GlcNAcα1→4Galβ1→R of the mucin MUC6 [107,108,109,110]. Thus, the lectin specificities of TFF1 and TFF2 seem to be different, but both could share GlcNAc as a common part of a complex ligand.

Furthermore, the frog integumentary mucins FIM-A.1 and FIM-C.1 from *Xenopus laevis* are capable of forming high-molecular-mass complexes probably via the lectin activities of their intrinsic multiple TFF modules [111]. In addition, the TFF modules in the sugar-degrading enzymes sucrase–isomaltase, α-glucosidase, and maltase–glucoamylase are also expected to have lectin activities [3]. Finally, the TFF modules in the human zona pellucida proteins ZP1 and ZPB would be perfectly designed to play a key role as lectins during fertilization; this could account for a species specificity.

Generally, the lectin activities of TFF peptides might explain diverse biological effects, such as their weak motogenic, (anti)-apoptotic, and angiogenic effects [108]. Theoretically, TFF peptides could activate a plethora of transmembrane glycoproteins by binding to their carbohydrate moieties, such as the receptors CXCR4 and CXCR7 [89,90], integrins [86], and CRP-ductin/DMBT1^gp340^ [86,87]. Furthermore, similar to galectins, TFF2 and homodimeric forms of TFF1 and TFF3 in particular could form two- and three-dimensional cross-linked lattices with various transmembrane glycoproteins, leading to supermolecular assembly and signal transduction [112,113].

### 2.2. Gastric TFF1 Mainly Occurs in a Monomeric Form with an Unusual Free Thiol Group: Possible Intracellular and Extracellular Functions in the Gastric Mucus Barrier and during Inflammation

Human gastric TFF1 occurs in different molecular forms, i.e., monomeric TFF1, homodimeric TFF1, and heterodimeric forms (TFF1-GKN2, TFF1-X/60k, TFF1-FCGBP) [103,114]. Generally, only minor amounts of gastric TFF1 are associated with the gastric mucus, as shown for humans [103,106] and *X. laevis* (ortholog xP1) [115]. This is even visible at the electron microscopic level, where TFF1/xP1 is localized in dense core regions of secretory granules and is not mixed with mucins [49,116]. However, dimeric TFF1 is capable of weakly binding in vitro to the mucin MUC6 [103].

Gastric TFF1 is also capable of forming a heterodimer with GKN2, as shown for humans and mice [45,100,103,106]. Of note, when gastric specimens are classically extracted (without SDS), TFF1–GKN2 is hardly detectable, particularly in the corpus [45,103]. Part of it seems to be soluble only in 1% SDS [45,103]. Thus, we postulated that TFF1–GKN2 might be a constituent of the inner insoluble layer of the gastric mucus [103]. This aspect is discussed further in Section 2.3.

Minute amounts of gastric TFF1 form a disulfide-linked heterodimer with FCGBP, as shown for humans and mice [45,103]. This is not surprising, as both TFF1 and FCGBP contain an odd number of cysteine residues and are synthesized in SMCs. The concentration of TFF1–FCGBP is higher in the antrum when compared with the corpus, as FCGBP is mainly expressed in the antrum of humans and mice [45,117]. The formation of the TFF1–FCGBP heterodimer is analogous, as described for TFF3–FCGBP in the intestine and saliva [101,102]. The postulated function of TFF1/3-FCGBP heterodimers in the innate immune defense is discussed in more detail in Section 2.4.

Systematic biochemical investigations revealed that, in gastric extracts, the major form of TFF1 is a secreted monomer with a free thiol group at Cys^VII^. This has been shown for *X. laevis* (ortholog xP1) [115], mice [45], and humans [103]. Normally, such free thiol groups act as a three-way switch, mediating assembly, retention, or degradation in the ER [118], but they are not secreted [95]. However, the unusual free thiol group in human TFF1 at Cys^VII^ is probably masked by four flanking acidic amino acids (see Table 2), and thus TFF1 mainly escapes assembly (dimerization), retention, or degradation and is predominantly secreted as a monomer. This explanation is based upon similar results obtained from Ig light chains, where flanking acid residues also allow the secretion of an unpaired cysteine [119]. Such flanking acid residues can drastically change the pKa of cysteine residues [120,121]. Of note, the unusual acid residues in TFF1/xP1 flanking Cys^VII^ are conserved in *X. laevis* (…PEC^VII^), mice (…QEEEC^VII^PF), and humans (…PPEEEC^VII^EF) [122]. It is expected that Cys^VII^ is exceptionally reactive (nucleophilic) because of the pronounced steric exposure outside the TFF domain and separation by proline residues [103]. Thus, it is hypothesized that TFF1, via its free thiol at Cys^VII^, has special intracellular as well as extracellular molecular functions.

Intracellularly, TFF1 is expected to be involved in the folding of cysteine-rich glycoproteins (such as the mucin MUC5AC), as in *Tff1*^KO^ mice the unfolded protein response (UPR) is activated [123]. For example, the expression of the protein disulfide isomerases (PDI) Pdia3/ERp57, Pdia4/ERp72, Pdia17/Agr2, and Grp78 is significantly elevated in *Tff1*^KO^ mice [45,123]. This led to the hypothesis that TFF1 plays a role in the ER protein folding machinery [123]. Thus far, the precise molecular function of TFF1 has not been elucidated. For example, it could have an intracellular function for the correct assembly of the mucin MUC5AC. Of note, recently a disulfide-linked TFF1 heterodimer (TFF1-X) with an Mr of about 60k was detected in humans [103], which hypothetically might represent a heterodimer with ERp57. ERp57 is capable of forming intermolecular disulfide bonds, e.g., with the major histocompatibility complex (MHC) class I [124], and is also known for its role in oncogenic transformation [125].

Extracellularly, the free thiol group of TFF1 has been proposed to act as a scavenger for reactive oxygen/nitrogen species (ROS/RNS) [45,103,115]. The formation of sulfur-centered radicals is a characteristic feature after the reaction of thiols with ROS [126,127]. As a consequence, sulfur-containing amino acids, such as cysteine, have a protective role against free radicals and heavy metals [128]. Protection from ROS/RNS is particularly important for the stomach, as oxidative stress plays a major role in stomach disorders [129]. Thus, TFF1 would be particularly well suited to protect the gastric surface mucous cells and probably also the adjacent population of highly proliferating precursor cells in the isthmus (review: [130]). The firmly adherent inner layer of the gastric mucus–bicarbonate barrier maintains a pH gradient with a nearly neutral pH at the mucosal surface [131]. This neutral pH allows the formation of thiolate anions and they are more apt to react chemically as a nucleophile with ROS/RNS [120,121,127]. Such a scavenger function for TFF1 is physiologically most important in the stomach, as here the extracellular level of ROS (e.g., H_2_O_2_ and O_2_^-^) is high due to dual oxidases (DUOX) and NADPH oxidases (NOX), respectively. DUOX plays a key role in innate mucosal immunity, preventing gastric colonization with bacteria [132,133]. Additionally, NOX enzymes are expressed in the gastric mucosa, the different forms being species-dependent [134]. Furthermore, the level of RNS can also be particularly high in the gastric lumen. After the reduction in exogenous nitrate (NO_3_^-^) from food to nitrite (NO_2_^-^) by saliva and microbiota, HO-NO forms in the gastric juice and disproportionates into NO [135], which is a highly reactive RNS-inducing S-nitrosylation of reactive cysteines [136,137]. In addition, NO can also react with O_2_^−^ forming peroxynitrite (ONOO^−^), which is the prototype of a toxic RNS [138]. Critical features for S-nitrosylation are acidic residues flanking the reactive cysteine [139], as well as copper ions [136]. Of special note, copper ions are known to bind to the glutamic acid residues flanking the Cys^VII^ of human TFF1 [140]. Taken together, TFF1 would be particularly well suited to protect the stomach from RNS.

The reactive Cys^VII^ of monomeric TFF1 could not only have intracellular and extracellular protective functions as a gastric tumor suppressor. TFF1 is also expected to play a protective role during inflammatory processes, as there is a fundamental connection to oxidative stress (“oxidative burst”) [137]. This assumption is supported by the observation that TFF1 is ectopically expressed during various chronic inflammatory conditions in humans [29,141], as well as in animal models of pancreatitis [31], asthma [34], and encephalitis [35], and in the murine spleen after *T. gondii* infection [36].

### 2.3. TFF2 is a MUC6-Binding Lectin: Function for the Stabilization of the Inner Insoluble Layer of the Gastric Mucus–Bicarbonate Barrier and More

In contrast to TFF1, gastric TFF2 is predominantly associated with mucins, as shown for humans [106], pigs [12], and *X. laevis* (ortholog xP4) [115]. Here, it binds to the mucin MUC6, but not MUC5AC [110]. Binding occurs as a lectin to the terminal oligosaccharide GlcNAcα1→4Galβ1→R in a Ca^2+^- and pH-dependent manner [107,108,109,110]. This explains why TFF2 and MUC6 are co-localized in the gastric mucus barrier [142]. The unusual αGlcNAc residue at the non-reducing terminal of the O-linked glycan of MUC6 is conserved from frog to human and is recognized by both the monoclonal antibody HIK1083, as well as the lectin GSA-II from *Griffonia simplicifolia* [143,144]. MUC6 is expressed early in vertebrate evolution, but was lost in teleost fishes [145]. This explains why even porcine TFF2 binds to *X. laevis* gastric mucin [115]. Of special note, α1,4GlcNAc-capped mucin-type O-glycans function as natural antibiotics against *H. pylori* infection by inhibiting the cholesterol α-glucosyltransferase of *H. pylori* [146,147]. That is probably one reason why *H. pylori* co-localizes with MUC5AC in the human stomach [148]. A key enzyme for the synthesis of the terminal αGlcNAc residue is α1,4-*N*-acetylglucosaminyltransferase (α4GnT); all mice lacking this enzyme (*A4gnt*^KO^) spontaneously develop antral adenocarcinomas through an inflammation-associated pathway [149]. Thus, αGlcNAc seems to serve as a gastric tumor suppressor.

A characteristic feature of human TFF2 is the N-glycosylation site (Table 2), which is lacking in the porcine and murine homologs. As a hallmark, human gastric TFF2 mainly contains the unusual N-linked monofucosylated *N,N’*-diacetyllactosediamine (LacdiNAc) oligosaccharide [150]. There is a dramatic diurnal variation of the TFF2 concentration in the gastric juice, with a maximum between 05:00 and 07:00, and N-glycosylation also varied diurnally, with a maximum between 17:00 and 23:00 [151]. The LacdiNAc moiety is also present in the gastric mucin MUC5AC and is recognized by the *H. pylori* adhesin LabA [152]. This is another reason why *H. pylori* mainly adheres to MUC5AC in the gastric mucus barrier.

TFF2 binding to MUC6 differs characteristically between human (mainly N-glycosylated TFF2) and pig (non-glycosylated TFF2). In porcine gastric mucus, the binding of TFF2 is much stronger than in human mucus and is even resistant to boiling in SDS [12,109,110]. This remarkable discrepancy cannot be explained by the differences in N-glycosylation, as murine Tff2, which is not N-glycosylated, behaves like human TFF2. Thus, it is assumed that it is rather the amino acid sequence which determines the strength of the TFF2 binding to MUC6. This view is also in agreement with recent results of the two *X. laevis* orthologs of TFF2, i.e., xP4.1 (N-glycosylated) and xP4.2 (non-glycosylated), where glycosylation has no influence on lectin binding to gastric mucin [115].

**Figure 2:**
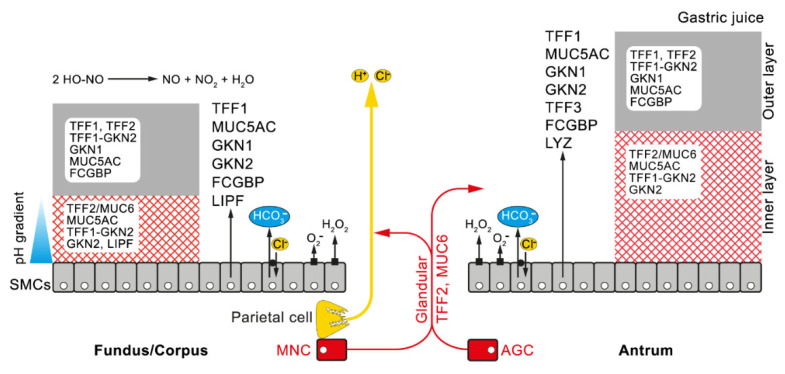
Model of the gastric mucus–bicarbonate barrier in the fundus/corpus and the antrum, respectively. The gastric mucus consists of an outer and an inner layer and originates from different cell populations, i.e., surface mucous cells (SMCs) and glandular cells (fundic mucous neck cells (MNCs) and antral gland cells (AGCs); shown in red). The SMCs in the fundus and corpus differ somewhat and they originate from different stem cells (review: [130]). Both SMC types synthesize TFF1, the mucin MUC5AC, and the gastrokines GKN1, and GKN2, whereas the fundic SMCs additionally secrete gastric lipase (LIPF), and the antral SMCs, lysozyme (LYZ), TFF3, and the major portion of Ig Fc binding protein (FCGBP) [117]. The MNCs and AGCs secrete TFF2 and the mucin MUC6 and the TFF2/MUC6 complex are expected to form the rather insoluble matrix of the inner layer. Further components of the inner layer are MUC5AC and probably TFF1–GKN2 and GKN2. The very hydrophobic LIPF is expected to be a component of the inner fundic mucus layer [117]. SMCs also secrete bicarbonate (HCO_3_^−^; shown in blue) in exchange with Cl^−^ creating a pH gradient along the inner layer. The dual oxidase (DUOX) in SMCs and the NOX enzymes generate H_2_O_2_ and O_2_^−^, respectively, at the apical surface, restricting microbial colonization (innate immune defense). The formation of NO in the gastric juice by the disproportionation of HO-NO is also shown.

Taken together, the highly specific lectin binding of TFF2 to a conserved O-linked oligosaccharide in MUC6 has implications for understanding the structure of the two-layered gastric mucus–bicarbonate barrier, which is composed of a loosely adherent (outer) layer and a firmly adherent, water-insoluble (inner) layer (Figure 2) [108,153]. 

The outer layer consists mainly of the mucin MUC5AC and only little MUC6 [110,153,154], and mixes with the gastric juice, which also contains TFF2 and mucins (mainly MUC5AC and only little MUC6) [151,155]. The adherent inner layer is composed of alternating layers of the mucins MUC5AC and MUC6 [154]. Here, the tight association of TFF2 and MUC6 is even visible by immunohistochemistry [142]. There are multiple indications that TFF2 stabilizes the water-insoluble inner layer physically [110]. For example, TFF2 affects the viscoelastic properties of mucous gels [156] and the *X. laevis* ortholog of TFF2 (xP4) prevents the shrinking of secretory granules of esophageal goblet cells during the processing of the samples for electron microscopy, due to its interaction with mucin [116]. Furthermore, *Tff2*^KO^ mice show accelerated progression of *Helicobaccter*-induced gastritis, as the inner mucus barrier is probably no longer very tight [55]. Thus, TFF2 and MUC6 are expected to form a mesh-like insoluble matrix (TFF2/MUC6 complex), where TFF2 may crosslink the MUC6 subunits in a Ca^2+^-dependent manner [109,110]. This matrix also creates an unstirred environment where neutralization by the secreted bicarbonate anions (HCO_3_^−^) occurs [131]. This nearly neutral pH in the inner layer also favors the lectin binding of TFF2 to MUC6, whereas an acidic pH in the outer layer favors the dissociation of TFF2 into the gastric juice [109]. Additionally, a large portion of TFF1–GKN2 and probably GKN2 are expected to be constituents of the inner layer, as these molecules are hardly soluble and *Gkn2*^KO^ mice have increased susceptibility to *H. pylori*-dependent immunopathology [103,157]. This model is depicted in Figure 2.

In contrast to human, pig TFF2 is also expressed in the pancreas. Here, TFF2 is clearly not associated with mucins and rather occurs in an unusual low-molecular homodimeric form, which is resistant to boiling in SDS [12]. This is probably due to the lack of MUC6 or of α4GnT in the porcine pancreas. Thus, porcine pancreatic TFF2 probably forms a complex with MUC6 only after having reached the pancreatic ducts and then the duodenum via Vater’s ampulla.

Finally, it will be a challenging goal for the future to identify the binding partner and to elucidate the molecular function of TFF2 in the lymphoid system, as *Tff2* deficiency influences the immune system [54]. It could well be that it also forms a matrix with MUC6 there.

### 2.4. TFF3 Mainly Forms a Disulfide-linked Heteromer with FCGBP: Postulated Function in the Innate Immune Defense

Human TFF1 and TFF3 are highly similar, as both consist of a single TFF domain and additionally contain a free Cys^VII^ residue (Table 2). However, as a hallmark, TFF1 and TFF3 differ, as TFF1 (in the stomach) is mainly not associated with the high-molecular-mass fraction after size exclusion chromatography [103,106], whereas TFF3 (in the intestine and saliva) is [101,102]. Here, TFF3 forms a disulfide-linked heterodimer with FCGBP, whose mature form consists of nearly 5400 amino acid residues, 435 being cysteine residues [101,158,159]. TFF3–FCGBP can easily form, as both TFF3 and FCGBP are secreted from the same cells, i.e., intestinal goblet cells and the mucous acini of salivary glands, respectively [159,160]. Furthermore, both TFF3 and FCGBP contain an odd number of cysteine residues. Based on quantitative measurements, it was suggested previously that probably only a single TFF3 molecule is linked to FCGBP, despite its enormous number of cysteine residues [101].

As shown for humans and mice, gastric TFF1 is also capable of forming a heteromer with FCGBP, but only to a very small extent [45,103]. Only little TFF1–FCGBP is generated, although both TFF1 and FCGBP are secreted from gastric SMCs. The reason might be the different reactivities of Cys^VII^ in TFF1 versus TFF3, as the flanking acidic residues are more numerous in TFF1 (see Table 2), which might prevent its heterodimerization with FCGBP. However, it is expected that the molecular function of TFF1–FCGBP (in the stomach) and TFF3–FCGBP (in the intestine, saliva, and many other mucous epithelia) is similar. 

Currently, the precise molecular function of FCGBP and its heteromers with TFF3 (TFF3–FCGBP) and TFF1 (TFF1–FCGBP) is not known. The repetitive glycoprotein FCGBP is ubiquitous in vertebrates and cephalochordates and it is secreted by most mucous epithelia from a variety of mucin-producing cells (similar as TFF3), and thus appears in the corresponding body fluids [145,159]. There are indications that FCGBP has a role in the innate immunity of mucous epithelia, where it is thought to regulate pathogen attachment and disease progression [161]. For example, FCGBP is an early response gene after microbial infection from fishes to humans [161,162] and it has also been hypothesized to act as a viral trap for HIV–antibody complexes [163]. Its expression is strongly induced by the TH2 cytokine interleukin-13 [164]. The multiple modular cysteine-rich domains of FCGBP might support the clearing of microorganisms or prevent bacterial infiltration through the mucus layer and even a number of bacterial proteins bear N-terminal domains homologous to the N-terminal domain of FCGBP [145]. The latter shows similarity to an enzyme from frog skin, which catalyzes the isomerization of L-amino acid residues into their corresponding D-enantiomers [165]. The heterodimerization of FCGBP with TFF3 (and also with TFF1) could synergistically enhance or modulate the binding of microorganisms due to the lectin activities of TFF1 and TFF3 [99]. Furthermore, TFF3–FCGBP could form a network by interacting with DMBT^gp340^, which is an agglutinin that also plays a general role in mucosal innate immunity [87,88]. For example, DMBT-1 aggregates *Streptococcus mutans* and *S. sanguis* [166]. The interaction could be lectin-based, similar to that of DMBT1 and galectin 3 [167]. Of note, FCGBP was also reported to be able to bind covalently to the mucin MUC2, probably as a component of the inner colonic mucus layer [168]. TFF3 may also play a role here. This might help to explain the phenotype of *Tff3*^KO^ mice in the DSS colitis model [60,169].

Other than playing a role in the innate immune defense of mucous epithelia, FCGBP could also fulfill a structural matrix function, as FCGBP seems to be expressed in the aorta, where it belongs to the most downregulated genes in patients with ruptured abdominal aortic aneurisms [170].

## 3. Conclusions and Clinical Perspectives

Taken together, within recent years, a body of evidence has been accumulated that TFF peptides fulfill diverse roles in the protection of mucous epithelia, other than acting solely as motogens (old paradigm). Their relatively high abundance in mucous epithelia does not fit with a predominant role as typical high-affinity ligands for receptors in mucous epithelia. However, even as low-affinity ligands, they can synergistically support mucosal repair, e.g., by restitution. In this case, the activation of certain glycosylated receptors, such as CXCR4 and CXCR7, might occur via the lectin activities of TFF peptides and would depend on the glycosylation of these receptors; the latter could vary in different cell types and would thus allow some specificity and diversity.

Currently, TFF peptides seem to have rather structural roles as part of the insoluble gastric inner mucus–bicarbonate barrier layer (TFF2/MUC6 complex) or as part of a postulated mucosal clearance/barrier system for different microbes (TFF3–FCGBP, TFF1–FCGBP). The latter might also be able to trap viruses with the help of immunoglobulins on mucosal surfaces. Both of these functions would indicate important roles of TFF2 and TFF3/TFF1 for the mucosal innate immune defense against pathogenic bacteria and viruses [171]. This is in line with the observation that TFF3 expression is regulated via TLR2 by commensal bacteria [84]. In contrast, the gastric tumor suppressor TFF1, with its free thiol group, could extracellularly protect the stomach from ROS and RNS. Furthermore, an intracellular function of TFF1 is still within the limits of expectation (folding of glycoproteins, such as MUC5AC, in the ER or even regulating gene expression). 

The function of TFF peptides in sites other than mucous epithelia is currently less well understood. Particularly, TFF1 might play a protective role during various inflammatory processes, even in the brain. TFF3 could also have a function during immune reactions in the CNS (expression in activated microglial cells). Furthermore, all three TFF peptides (especially TFF3) could also act as neural chemotactic factors, as they show developmental changes, particularly in the cerebellum [19]. In contrast, TFF2 seems to play a special role in the immune system, maybe as part of a matrix.

Based on these diverse molecular functions of TFF peptides, interesting clinical applications might arise in the future [172]. For example, TFF1, as well as TFF3, have been used to prevent oral mucositis after different regimes of chemotherapy [173,174]. Furthermore, the pharmacological inhibition of the TFF3 homodimer by monomerization via a synthetic drug has been reported to reduce the growth of colorectal carcinoma in vivo [175]. There are many more applications possible, as microbiota probably play a pivotal role in the development and severity of chemotherapy-induced mucositis [176]. For example, TFF1–FCGBP, as well as TFF3–FCGBP, could be components of new anti-microbial formulations, e.g., used as artificial saliva. It could be also tested whether synthetic peptides mimicking the C-terminal of TFF1, including the activated Cys^VII^, can prevent *Tff1*^KO^ mice from developing antral carcinomas.

In a further attempt, the region in TFF modules responsible for lectin activities could be defined in detail. In combination with theoretical modeling methods, a mutagenesis program could create variants with new/novel affinities for different carbohydrate moieties. Such a program should be greatly facilitated by the fact that biologically active TFF peptides can be generated, not only by recombinant technologies, but also pure chemical synthesis, where even the correct formation of disulfide bridges is possible [103,105]. 

Taken together, TFF peptides appear to share characteristic features with the multifunctional family of galectins [177]. For the future, many interesting medical applications concerning TFF modules can be expected, particularly for treating carcinomas and mucosal infections, as well as for supporting chemotherapy.

## Figures and Tables

**Table 1:** Expression sites of human TFF peptides.

TFF Peptides	Exocrine Secretion (Major/Minor)	Endocrine Secretion
TFF1	**Stomach**, conjunctiva, lacrimal apparatus, salivary glands, lung, urogenitary tract	CNS
TFF2	**Stomach, Brunner’s glands**, salivary glands	CNS, lymphoid tissues
TFF3	**Intestine, salivary glands,** **lung, uterus, vagina**, conjunctiva, lacrimal apparatus, inner ear, esophagus, stomach, gallbladder, Vater’s ampulla, urinary tract	CNS, thyroid, lymphoid tissues, endocrine pancreas

Major expression sites are shown in bold.

**Table 2:** Structures and natural forms of human TFF peptides at their predominant expression sites.

TFFs	Structures	Expression Sites	Natural Forms (Major/Minor)
TFF1	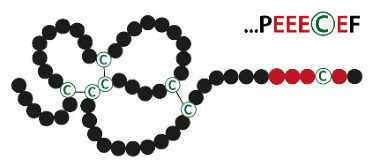	Stomach	**TFF1_mono_** TFF1-FCGBP TFF1-X (60 k) TFF1-GKN2 TFF1-TFF1
TFF2	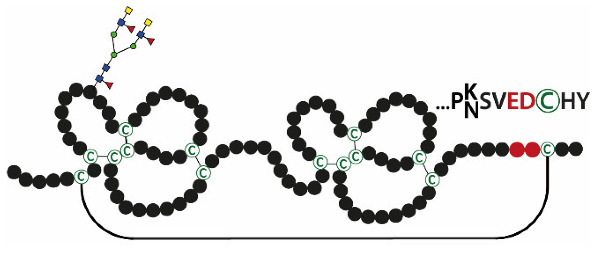	Stomach	**TFF2/MUC6** TFF2
TFF3	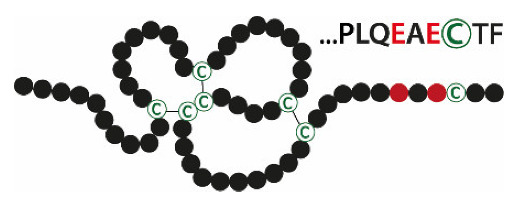	Intestine, salivary glands	**TFF3-FCGBP** TFF3-TFF3 TFF3_mono_

Acidic residues flanking the C-terminal cysteines are shown in red. For TFF2, the N-linked carbohydrate moiety is depicted (yellow squares, GalNAc; blue squares, GlcNAc; green circles, Man; red triangles, Fuc). Major natural TFF forms are shown in bold.

## Abbreviations

DUOX	Dual oxidase
ER	Endoplasmic reticulum
FCGBP	IgG Fc binding protein
GKN	Gastrokine
NOX	NADPH oxidase
SDS	Sodium dodecyl sulfate
SMC	Surface mucous cell
TFF	Trefoil factor family
